# Impact of male alternative reproductive tactics on female costs of sexual conflict under variation in operational sex ratio and population density

**DOI:** 10.1002/ece3.3631

**Published:** 2017-12-02

**Authors:** Erica Jeffery, Alex Córdoba‐Aguilar, Bernard Roitberg

**Affiliations:** ^1^ Simon Fraser University Burnaby BC Canada; ^2^ Departamento de Ecología Evolutiva Instituto de Ecología Universidad Nacional Autónoma de México Coyoacan Mexico

**Keywords:** density‐dependent, male alternative reproductive tactics, OSR‐dependent, sexual conflict

## Abstract

Sexual conflict over mating rate is both pervasive and evolutionarily costly. For females, the lifetime reproductive fitness costs that arise through interactions with potential mates will be influenced by the frequency of such interactions, and the fitness cost of each interaction. Both of these factors are likely to be influenced by variation in operational sex ratio (OSR) and population density. Variation in OSR‐ and density‐dependent male alternative reproductive tactics (ARTs) may be particularly important if the fitness costs that females experience vary with the reproductive tactics that males express. Using a simple model, we consider several examples of OSR‐ and/or density‐dependent variation in male ARTs and the frequency of male–female interactions, and find that variation in the expression of male ARTs has the potential to augment or diminish the costs of frequent male interactions for females. Accurately documenting variation in the expression of male ARTs and associated female fitness costs will benefit future work in this area.

## 1 INTRODUCTION

Sexual reproduction often entails conflict, as reproductive partners frequently differ in their evolutionary interests (Parker, 1979). One area where such conflict is widely observed and has been greatly investigated is mating rate; whereas females may require relatively few matings to maximize fitness, in many species a male's fitness increases with his mating frequency (Andersson, 1994; Arnqvist & Rowe, 2005; Chapman, Arnqvist, Bangham, & Rowe, 2003; Gavrilets, Arnqvist, & Friberg, 2001; Parker, 2006). For example, in the case of two previously mated individuals encountering one another, selection may favor a male that remates, but a female that does not (Parker, 1979). The outcome of such an interaction can result in both direct and indirect fitness costs or benefits for the male or the female (Holland & Rice, 1998; Parker, 1979, 2006). Direct fitness costs to females resulting from conflict interactions have been of particular empirical and theoretical interest. Such costs can result in selection for traits that allow females to avoid direct fitness costs when interacting with males, and can therefore be drivers of evolutionary change (Clutton‐Brock & Parker, 1995; Parker, 1979, 2006; Rowe, Arnqvist, Sih, & Krupa, 1994). Furthermore, because the mean reproductive successes of males and females are necessarily equivalent in a population with a 1:1 sex ratio, direct fitness costs to females will also limit male fitness, and can depress the fitness of the entire population (Rankin, Dieckmann, & Kokko, 2011).

Intrasexual reproductive competition may select for more than one alternative to obtaining fertilizations. When expressed as discrete phenotypes in either males or females, these alternatives are better known as alternative reproductive tactics (ARTs) (Gross, 1996; Brockmann, 2001; reviewed by Shuster & Wade, 2003; Taborsky, Oliveira, & Brockmann, 2008). Alternative reproductive tactics are expected to evolve whenever they allow individuals to increase their fitness beyond what could be achieved by conventional tactics, and examples of male ARTs are plentiful (reviewed in Oliveira, Taborsky, & Brockmann, 2008). Prior to copulation, male ARTs may take the form of territorial versus satellite and/or floating individuals, courting versus noncourting, guarding versus searching, or bourgeois versus parasite individuals, among others (Oliveira et al., 2008). As alternative solutions to the problem of obtaining female mates, it is conceivable that male ARTs could entail different fitness costs for females. For example, water strider males exhibit both courtship and coercion in pursuing mates, the former posing fewer direct costs to females than the latter (Arnqvist, 1997). The implication of such differences in female's costs for systems with intraspecific variation in male ARTs has not been explored until recently (Alonzo, 2008; Reichard, Le Comber, & Smith, 2007; Weir, 2012). Explicit consideration of such effects will help increase our understanding of the impact of sexual conflict on individuals and populations.

Two factors known to influence the expression of male ARTs are operational sex ratio (the ratio of potentially receptive males to receptive females at any one time, OSR; Emlen, 1976; Jirotkul, 1999a) and population density (Jirotkul, 1999b; Rowe et al., 1994; Tomkins & Brown, 2004). Although efforts are frequently made to examine the independent effects of OSR and population density on mating behavior (Alonso‐Pimentel & Papaj, 1996; Jirotkul, 1999a,b; de Jong, Wacker, Amundsen, & Forsgren, 2009; Wacker et al., 2013), together the two determine the encountered number of potential competitors and potential mates (Emlen & Oring, 1977; Kokko & Rankin, 2006), and thus both influence perceived levels of mate competition and mate availability. Actual relationships between OSR or population density and the frequency of male ARTs may be complex, and will vary from system to system (Aronsen et al., 2013; Knell, 2009; Kokko & Rankin, 2006; Weir, Grant, & Hutchings, 2011). Furthermore, OSR and population density are unlikely to vary completely independently of one another. Not only are the two metrics linked to one another through numbers of individuals; it is also possible that individuals will respond to one factor in such a way that it induces change in the other (e.g., secondary sex ratio variation in response to increased population density in red deer; Kruuk, Clutton‐Brock, Albon, Pemberton, & Guinness, 1999). Whatever the underlying mechanism, given the extent to which varying sex ratios and population densities result in temporal and spatial variation in the expression of male ARTs, we should expect similar variation in any female fitness costs associated with these two factors.

Increased male‐encounter rates due to high population densities and/or male‐biased sex ratios are expected to increase female fitness costs associated with remating and/or resistance, and decrease the marginal benefits of remating for females (Härdling & Kaitala, 2005; Kokko & Rankin, 2006). However, if the expression of density‐ and OSR‐dependent male ARTs alters the cost of different male interactions for females, then there may also be an indirect relationship between population‐level factors and direct fitness costs to females. Although several empirical studies have examined female fitness costs relating to male ARTs (Johnson & Brockmann, 2010; Reichard et al., 2007; Watters, 2005), few have considered these costs in conjunction with OSR‐ and/or density‐dependent effects (Weir, 2012) or the compounded effects of mate‐encounter rate on female fitness. We introduce here a theoretical framework that we hope will assist in further investigations of the relationships between population states, mating behavior, and female costs. We present a simple model linking OSR and population density to the number of costly male interactions experienced by an individual female, and then examine the resulting female conflict costs in a two male ARTs system under a variety of parameter values. In particular, our model distinguishes potential quantitative effects (variation in the number of male encounters) from qualitative effects (variation in the type of male behavior encountered). We consider this model an important first step toward a better understanding of how variation in population‐level factors can influence coevolutionary dynamics between males and females; future research will need to investigate the ways in which selection on females could impact male ARTs, under such variation in conditions.

## 2 THE MODEL

### 2.1 Female fitness costs

For the purposes of our model, we assume that a female experiences some net direct fitness cost (or benefit) as a result of each interaction she has with a male, and that those costs accumulate in an additive fashion over the course of a given time period (e.g., the reproductive season). These costs are considered to be direct fitness costs to the female in that they decrease her lifetime reproductive success, either through decreasing her lifespan, and/or through decreasing her net reproductive output. If males express different alternative reproductive tactics, and subsequently differ in the direct fitness costs they impose on females, we can weight those costs by how often females encounter different male tactics. The net fitness cost of male–female interactions for the female during that time period, then, is given by

(1) $$C_{f}=\sum_{i=1}^{T}c_{i}\times n_{i}$$

where *c*_*i*_ is the mean net female fitness cost arising from an interaction with a male expressing tactic *i* (i.e., the male‐interaction cost), *n*_*i*_ is the number of interactions the female has with a male (or males) expressing tactic *i* during the time period, and *T* is the total number of male tactics in the system. It should be noted that here we are interested in quantifying direct costs to females due to interactions with males. In practice, a female who encounters zero males during the current time period (and who has no access to viable sperm stored from encounters during previous time periods) will experience essentially infinite costs due to an inability to reproduce. Such a female would also experience zero male‐interaction costs, however. Male‐interaction costs may be any real number; we have chosen to represent female fitness costs using positive values in order to make them easier to visualize. Therefore, if the female experiences a net fitness benefit as a result of interacting with a male instead of a net fitness cost, *c*_*i*_ would be <0.

If we assume that the probability that a female encounters and interacts with a particular male is independent of the tactic he is expressing (i.e., the probability of interacting with a female_*i*_ is the same for all males), then equation (1) may be restated as:

(2) $$C_{f}=\left(\sum_{i=1}^{T}c_{i}m_{i}\right)\times n$$

where *m*_*i*_ is the proportion of males that express tactic *i*, and *n* is the total number of male interactions that the female experiences during the time period of interest. For a system in which male reproductive tactics are plastic rather than fixed, this is equivalent to assuming that each time a female encounters and interacts with a male, he expresses one of the existing tactics with some known probability (*m*_*i*_). In a system with two male reproductive tactics (tactic 1 and tactic 2), equation (2) may be restated as:

(3) $$C_{f}=\left(c_{1}m_{1}+c_{2}\left(1-m_{1}\right)\right)\times n.$$

### 2.2 Population density‐ and OSR‐dependent variation in female fitness costs

As long as male‐interaction costs (*c*_*i*_) remain constant, *m*_*i*_ and *n* are the only factors in our model that contribute to variation in female fitness costs (*C*_f_). The assumption that male–female interaction rates and the expression of male ARTs are independent of one another is useful because it allows us to consider the impact of each of these factors on female fitness costs separately. For all of the following examples, we use equation (3) to calculate female fitness costs for a two‐tactic system. First, we consider a situation in which the number of male interactions experienced by a female during a given time period is constant, but the relative number of males expressing a particular male alternative reproductive tactic varies in response to population‐level parameters. There are many possible ways in which the expression of male ARTs could vary with either population density or OSR; a recent meta‐analysis by Weir et al. (2011) outlines several examples of generally observed OSR‐dependent variation in male reproductive tactics. It should be noted that the patterns of male behavior Weir et al. (2011) observed were based on variation in the frequency (rates and counts per sample) of a particular male behavior, whereas here we are concerned with variation in the proportion of males expressing one male reproductive tactic instead of another (e.g., *m*_1_ vs. *m*_2_). If the prevalence of a particular male behavior is measured as a per‐opportunity frequency (i.e., per‐female interaction, or per‐available female), the two metrics are functionally equivalent; otherwise, equating observed frequencies of male behavior with male propensity to engage in a behavior may be misleading (de Jong, Forsgren, Sandvik, & Amundsen, 2012). Note that, if the probability of male–female interaction differs for different male tactics equation (1) should be used for estimating net fitness costs for females instead of equation (2).

For the purposes of illustration, we will compare four possible patterns of expression in male reproductive tactics, assuming in each case that the male‐interaction cost associated with the first tactic is twice that of the second (i.e., *c*_1_ = 1; *c*_2_ = 0.5):

- Expression of male tactic 1 is constant
- Expression of male tactic 1 increases with OSR
- Expression of male tactic 1 decreases with OSR
- Expression of male tactic 1 reaches its maximum at intermediate levels of OSR

**Figure 1:**
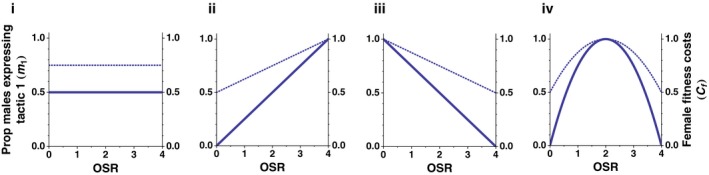
OSR‐dependent variation in expression of male ARTs, and related female fitness costs. Solid line represents proportion of males expressing alternative tactic 1 (*m*_1_); the proportion of males expressing alternative tactic 2 (not shown) is the inverse of this curve. i, The expression of male tactic 1 does not vary with OSR; ii, expression of male tactic 1 increases with OSR; iii, expression of male tactic 1 decreases with OSR; iv, expression of male tactic 1 reaches its maximum at intermediate levels of OSR. The dotted line shows resulting female fitness costs as a function of OSR, when the male‐interaction cost associated with tactic 1 is twice than of tactic 2 (*c*_1_ = 1, *c*_2_ = 0.5), and when females interact with one male during the time period of interest (*n* = 1)

Figure 1 illustrates each of these four patterns of variation in the proportion of males expressing tactic 1 (*m*_1_) versus tactic 2 (*m*_2_), for a two‐tactic system. In order to facilitate comparison between scenarios ii–iv, in each of these cases, *m*_1_ ranges from 0 to 1 for the values of OSR shown (solid lines). The dotted lines in Figure 1 show corresponding variation in net female costs for each of the scenarios when females interact with one male during the time period in question (i.e., *n* = 1). In all four scenarios, variation in the expression of the two male alternative tactics is unrelated to variation in population density; female fitness costs are therefore also independent of variation in population density. The Mathematica (Wolfram Research, Champaign, IL) code used to create these and all other figures in this paper can be found in Appendix S1 (Appendix S2 is an editable doc. file).

Next, we will consider what happens when the number of male–female interactions varies. Modeling male–female interaction and/or encounter rates realistically can be a complicated endeavor (Hutchinson & Waser, 2007). In addition to determining male and female densities, OSR and population density may also affect population spatial structure, mate searching behavior, and individual movement patterns (Kokko & Rankin, 2006), making the relationship between male–female interaction rates and population parameters complex. For the sake of simplicity, we will consider situations in which number of interactions varies as a linear function of either population density, or operational sex ratio:

- The number of of male–female interactions varies with population density. In some systems, the number of male–female interactions may be limited by opportunity for male–female encounters, which in turn may be proportional to population density. The number of males a female interacts with during a given time period could then be expressed as some function of male density:
  (4) $$\rho_{m}=\rho \times \frac{\text{OSR}}{\text{OSR}+1}$$
  and
  (5) $$n=\rho_{m}\times r\times k+b$$
  where ρ_m_ is male density (the total number of reproductively active males per unit area, during the current time period), ρ is the population density (the total number of reproductively active individuals, both males and females), and OSR is the operational sex ratio (males:females). Here, we assume that the number of males with which a female interacts depends upon both male density, and on the size of her range, *r*, in unit area. Parameters *k* and *b,* respectively, describe the slope and intercept of the assumed linear relationship between *n* and ρ_m_. These and all other model parameters used in this paper are summarized in Table 1.

**Table 1:** Summary of model parameters

Parameter	Definition
*C*_f_	Net female fitness cost associated with male interactions, accrued by an individual female during the time period of interest
*c*_*i*_	Mean net female fitness cost arising from an interaction with a male expressing tactic *i* (i.e., the male‐interaction cost)
*n*_*i*_, *n*	Number of interactions the female has with males expressing tactic *i*, or total number of interactions with all males, during the time period of interest
*T*	Total number of male tactics in the system
*m*_*i*_	Proportion of males expressing tactic *i* (or the probability that a male will express tactic *i*)
ρ, ρ_m_	Population density and male density: the average number of reproductively active males and females (ρ), or males only (ρ_m_), per unit area
*r*	Size of an individual female's range measured in units area
OSR	Operational sex ratio: the ratio of reproductively active males to reproductively active females
*k*,* b*	Slope and intercept, respectively, of hypothetical linear relationships between parameters

**Figure 2:**
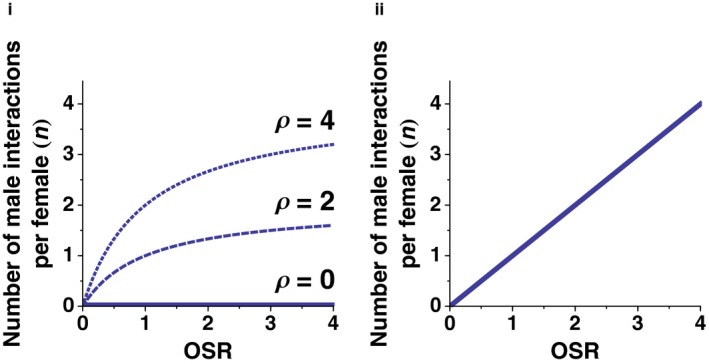
Number of male interactions per female as a function of OSR and population density. Operational sex ratio (OSR) is measured as the number of reproductively active males per reproductively active female; population density (ρ) is measured as the total number of reproductively active males and females per unit area. i, Number of male–female interactions is a function of male density (ρ_m_), and therefore varies with both OSR and population density: *n* = ρ_m_ × *r* × *k* + *b*, where *r*,* k* and *b* are constants (here, *r* = 1, *k* = 1, and *b* = 0). ii, Number of male–female interactions is a function of OSR, and is independent of population density: *n* = OSR × *k* + *b*, where *k* and *b* are constants (as above, *k* = 1 and *b* = 0)

According to equations (4) and (5), the relationship between number of male–female interactions and OSR is nonlinear; Figure 2i illustrates how *n* varies as a function of OSR and density for three different population densities, assuming *r* = 1 unit area, *k* = 1, and *b* = 0.

- The number of male–female interactions varies with OSR. Even if the opportunity for male–female encounters is not limited by male density, sex ratios may impact the number of male–female interactions. For example, males may divide their attention between available females, making the number of male interactions per female proportional to the number of males per female (i.e., OSR):
  (6) n=OSR×k+b
  where *k* and *b* describe the assumed linear relationship between the number of male interactions per female (*n*) and OSR. Figure 2ii shows this relationship for values of *k* = 1 and *b* = 0.

### 2.3 Combining ARTs and population drivers

**Figure 3:**
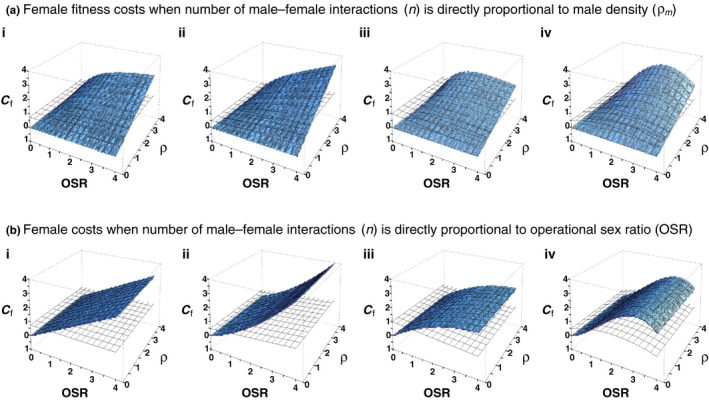
Variation in female fitness costs associated with OSR‐dependent male ARTs. The solid surfaces represent female fitness costs when the number of male interactions per female is directly proportional to (a) male density (ρ_m_; see Figure 2i), or (b) the operational sex ratio (OSR; see Figure 2ii). The relationships between the expression of male ARTs and OSR correspond to those shown in Figure 1: i, the expression of male tactic 1 does not vary with OSR (*m*_1_ = *m*_2_ = 0.5); ii, expression of male tactic 1 increases with OSR; iii, expression of male tactic 1 decreases with OSR; iv, expression of male tactic 1 reaches its maximum at intermediate levels of OSR. Female fitness costs associated with the first male tactic are assumed to be twice that of the second (*c*_1_ = 1; *c*_2_ = 0.5). The transparent surfaces represent female fitness costs associated with male ARTs when the number of male–female interactions is independent of either OSR or population density and constant (*n* = 1); these surfaces are therefore identical within each column of the figure. Dotted lines in Figure 1i–iv show the two‐dimensional equivalent of these transparent surfaces

The combined effects of (a) male response to OSR and (b) the population‐level determinants of male–female interaction rates on female fitness costs can be seen in Figure 3. Here, we apply the four patterns of variation in male ARTs (described in i, ii, iii, and iv) to situations with either density and/or OSR‐dependent male–female interaction rates. This yields eight surfaces representing variation in direct, net fitness costs to females. For comparison, the transparent surfaces in each plot show female fitness costs when the number of male–female interactions is independent of either OSR or population density (*n* = 1). These surfaces vary between columns (i.e., based on variation in male ARTs), but are identical within each column; the transparent surfaces are the three‐dimensional equivalent of the dotted lines shown in Figure 1. Several features stand out:

First, in so far as they increase the number of male–female interactions, increases in population density and/or OSR lead to increases in direct, net fitness costs for females. However, where changes in OSR result in both qualitative changes in male–female interactions (due to variation in male ARTs) as well as quantitative changes (i.e., in the number of interactions), the relationships between OSR and direct female fitness costs are nonlinear (Figure 3ii–iv). This is the case whether the number of male–female interactions is proportional to population density (Equation (5)) or OSR (Equation (6)), because OSR also influences the latter through its impact on male density (Figure 2).

Second, although variation in female costs is congruent with variation in male tactics (again comparing the transparent surfaces in Figure 3 with the expression of male ARTs shown in Figure 1), quantitative variation in the number of male interactions per female has a strong impact on the scale of female fitness costs. For example, in Figure 3aii, even when the majority of males are expressing the more costly tactic (i.e., OSR > 2), costs to females remain low as long as population densities (and therefore number of male–female interactions) also remain relatively low. Similarly, in Figure 3aiii, the majority of males are expressing the less costly tactic when OSR > 2, yet female costs are still relatively high as long as population density is also high. These results are based on a relatively moderate ratio between male‐interaction costs (i.e., *c*_1_ = 1 and *c*_2_ = 0.5). As the relative difference of male‐interaction costs increases, so will the impact of variation in expression of male ARTs on net female fitness costs. Nonetheless, such effects will be most apparent when the number of male–female interactions is high rather than low.

Third, whether we choose to calculate the number of male–female interactions based on male density or OSR will have strong qualitative effects on net female fitness costs. If male–female interaction rates are density‐dependent, low population densities will have a moderating effect on the costs females experience as a result of male ARTs. If male–female interaction rates are instead a function of OSR, females can experience high fitness costs even when male densities are low, as long as OSR is high.

Fourth, we have assumed that interactions with males will have a net fitness cost for females, whereas such interactions might instead be beneficial (e.g., due to nuptial gifts, or other benefits of polyandry). Thus, the sign for *c*_*i*_ could be positive or negative. How exactly these differences might play out in the real world is not clear, but we would not expect the resultant fitness surfaces to necessarily mirror one another due to the fact that fitness benefits and costs can be bounded differently from one another. For example, benefits from serial nuptial gifts may be saturating (i.e., nonadditive), whereas energetic costs from serial harassment may be linear (or asymptote at a far greater distance from the origin than the former). Investigating the nature of variation in female fitness benefits versus costs in relation to OSR‐ or density‐dependent male ARTs is a potentially fruitful topic for future theoretical work.

Finally, we can ask what this modeling exercise might tell us about mating conflict in theory and in nature. If we were to construct a full ESS, two‐sex model, it is clear that the use of simple, context‐free female‐cost constants could be problematic. We have shown that such costs can vary from near zero to very high depending upon population values. The surfaces that we have deduced are only moderately complex, however, which suggests that simple functions could easily replace those worrisome constants. Given that our approach provides estimates of direct, net fitness costs to females across a gradient of male tactics, we can now also estimate fitness costs (and benefits) that might arise if female reproductive tactics were to evolve, and subsequent evolutionary responses by males that could move the population to a different position along the gradient.

In terms of empirical studies of mating conflict, our model illustrates the potential significance of variation in both the expression of male ARTs and the number of male–female interactions when it comes to estimating the impact of OSR‐dependent male ARTs on female fitness. If a male ART that imposes direct fitness costs on females is more prevalent when male–female interaction rates are high (e.g., at a high OSR or high population density), females will experience higher male interaction related fitness costs than predicted by male‐interaction rates alone. If the reverse is true (i.e., the male ART that is associated with higher male‐interaction costs for females is less frequent at a high OSR/population density), then plasticity in male reproductive tactics could in fact mitigate the cost of frequent male–female interactions for females. Our model suggests that the relative magnitude of the male‐interaction costs associated with the different male reproductive tactics would influence overall patterns of variation in female fitness costs, whereas absolute differences in male‐interaction costs would determine the scale of those costs. For any pattern of OSR‐dependent variation in male ARTs (e.g., Figure 1), if the ratio of *c*_1_:*c*_2_ is held constant, the relative difference between net female fitness costs at any two OSR (or population densities) will also remain constant. The greater the relative difference in male‐interaction costs, the more abrupt the variation in net female fitness costs as the relative representation of the two male tactics changes. As the absolute magnitude of male‐interaction costs increases, so will net female fitness costs.

In our model, we consider only the potential for female costs to arise out of male–female interactions. If such costs exist, females may be under selection to express OSR‐ or density‐dependent tactics that reduce the fitness costs of interacting with males (Parker, 2006). For example, females may become more aggressive toward males when operational sex ratios are high, or may avoid interacting with males under high‐density conditions by altering their spatial (e.g., residence time) and/or temporal (e.g., emergence) overlap. Sex ratio and population density may also impact female fitness via female densities, and the costs of intrasexual competition could offset or outweigh male‐interaction‐related fitness costs for females (Smith, 2007; Smith & Sargent, 2006). Finally, males may also experience divergent fitness costs when they express different reproductive tactics (Christenson & Goist, 1979; Gross, 1996; Lucas & Howard, 1995; Smith, Schrank, & Brockmann, 2013; Vahed, 2007). Differential fitness of males and females could potentially alter OSR and population density in the next reproductive time frame, leading to carry over effects of population‐level variation.

## 3 CONCLUSION

We have presented a simple model examining the potential effects of variation in OSR and population density on female fitness costs due to interactions with males. Specifically, we investigate how differences in the female fitness costs associated with male alternative reproductive tactics might interact with variation in the frequency of male–female interactions. Other work has drawn attention to the importance of considering both variation in the probability of particular male reproductive behaviors, as well as variation in the opportunity for behavior (i.e., male–female encounter rates), when interpreting observed frequencies of male reproductive tactics (de Jong et al., 2012). Here, we show that variation in probability and opportunity can have an important impact on female fitness, because together they determine number of costly interactions. These predictions are for the most part intuitive, and provide a clear demonstration of how fitness costs due to one‐on‐one interactions can be greatly mitigated or exacerbated by existing population factors.

Despite the intuitive nature of our model, we conclude by highlighting some issues that may not be obvious: (1) OSR can impact costs to females by impacting both expression of ARTs and the number of interactions with males, resulting in nonlinear cost functions and (2) male‐interactions costs to females are not monotonic with regard to OSR. If males adjust to the less costly ART with increasing OSR, female fitness costs will maximize at some intermediate value of OSR even if interaction rates increase (e.g., Figure 3biii).

## AUTHOR CONTRIBUTION

All three authors conceived the work. EJ and BR structured the model. All authors interpreted, drafted and revised the work critically for important intellectual content.

## CONFLICT OF INTEREST

The authors declare no conflict of interest.
