# Supplementary material for: Impact of male alternative reproductive tactics on female costs of sexual conflict under variation in operational sex ratio and population density
Source: Ecol Evol. 2017 Dec 2;8(1):584–91. doi: 10.1002/ece3.3631 (PMC5756886; doi:10.1002/ece3.3631)
Supplement: Supplementary file 2 [file ECE3-8-584-s002.docx]

**Online Appendix A. Mathematica code for figures in Jeffery et al., “Impact of Male Alternative Reproductive Tactics on Female Costs of Sexual Conflict Under Variation in Operational Sex Ratio and Population Density”**

**Figure 1. OSR-dependent variation in expression of male ARTs, and related female fitness costs. Solid line represents proportion of males expressing alternative tactic 1 (m_1_); dotted line indicates proportion of males expressing alternative tactic 2 (m_2_).**

**Solid line represents proportion of males expressing alternative tactic 1 (m_1_) ; proportion of males expressing alternative tactic 2 (m_2_) can be visualized as the inverse of m_1_.**

"Clear all previous definitions:";

Clear["Global`*"]

"Define global x- and y-axes for all Figure 1:";

xticks=Join[Table[{x,NumberForm[x,4],{0,0.02}},{x,0,4,1}],Table[{x,"",{0,0.01}},{x,0,4,0.5}]];

yticks=Join[Table[{y,NumberForm[y,{4,1}],{0,0.02}},{y,0,1,0.5}],Table[{y,"",{0,0.01}},{y,0,1,0.1}]];

"Define global figure format for all Figure 1:";

SetOptions[Plot,BaseStyle->{FontFamily->"Helvetica",FontWeight->"Bold",FontSize->22},PlotRange->{{0,4},{0,1.05}},Axes->True,Frame->{True,True,False,True},FrameTicks->{xticks,yticks,None,yticks},FrameTicksStyle->Directive[18],FrameLabel->{"OSR","Prop males expressing

tactic 1 (m_1_)",None,"Female fitness costs

(C_f_)"},ImagePadding->95,AspectRatio->1,ImageSize->400];

i) The expression of male tactic 1 does not vary with OSR (m_1_= m_2_= 0.5).

"Proportion of males expressingt tactic 1 = proportion of males expressingt tactic 2 = 0.5";

m1i=0.5;

m2i=1-m1i;"Proportion of males expressing tactic 1 + tactic 2 = 1";

"Female fitness costs associated with male interactions:";

c1=1;"c_1_ = female fitness cost associated with alternative male reproductive tactic 1";

c2=0.5;"c_2_ = female fitness cost associated with alternative male reproductive tactic 2";

"Male-female interaction rates:";

n=1;"Number of male-female interactions is constant = 1";

"Response curve for male tactic A (solid line):";

plotm1i=Plot[m1i,{OSR,0,4},PlotStyle->{Thickness[0.02]}];

"Female cost curve when frequency of male-female interactions is constant = 1 (transparent surface):";

plotci=Plot[(c1*m1i+c2*m2i)*n,{OSR,0,4},PlotStyle->{Thickness[0.012],Dotted}];

"Show response curves for both male reproductive tactics in one plot:";

Show[plotm1i,plotci]

**ii) Expression of male tactic 1 increases with OSR (i.e., positive linear response curve).**

"Define positive linear curve for male tactic 1, and inverse for male tactic 2:";

k=0.25;"k = slope";

b=0;"b = intercept";

m1ii=Clip[(k*OSR+b),{0,1}];"Clip function limits proportion of males expressing ART to 0–1";

m2ii=1-m1ii;"Proportion of males expressing tactic 1 + tactic 2 = 1";

"Female fitness costs associated with male interactions:";

c1=1;"c_1_ = female fitness cost associated with alternative male reproductive tactic 1";

c2=0.5;"c_2_ = female fitness cost associated with alternative male reproductive tactic 2";

"Male-female interaction rates:";

n=1;"Number of male-female interactions is constant = 1";

"Response curve for male tactic 1 (solid line):";

plotm1ii=Plot[m1ii,{OSR,0,4},PlotStyle->{Thickness[0.02]}];

"Female cost curve when frequency of male-female interactions is constant = 1 (transparent surface):";

plotcii=Plot[(c1*m1ii+c2*m2ii)*n,{OSR,0,4},PlotStyle->{Thickness[0.012],Dotted}];

"Show response curves for both male reproductive tactics in one plot:";

Show[plotm1ii,plotcii]

**iii) Expression of male tactic 1 decreases with OSR (i.e., negative linear response curve).**

"Define negative linear curve for male tactic 1, and inverse for male tactic 2:";

k=-0.25;"k = slope";

b=1;"b = intercept";

m1iii=Clip[(k*OSR+b),{0,1}];"Clip function limits proportion of males expressing ART to 0–1";

m2iii=1-m1iii;"Proportion of males expressing tactic 1 + tactic 2 = 1";

"Female fitness costs associated with male interactions:";

c1=1;"c_1_ = female fitness cost associated with alternative male reproductive tactic 1";

c2=0.5;"c_2_ = female fitness cost associated with alternative male reproductive tactic 2";

"Male-female interaction rates:";

n=1;"Number of male-female interactions is constant = 1";

"Response curve for male tactic A (solid line):";

plotm1iii=Plot[m1iii,{OSR,0,4},PlotStyle->{Thickness[0.02]}];

"Female cost curve when frequency of male-female interactions is constant = 1 (transparent surface):";

plotciii=Plot[(c1*m1iii+c2*m2iii)*n,{OSR,0,4},PlotStyle->{Thickness[0.012],Dotted}];

"Show response curves for both male reproductive tactics in one plot:";

Show[plotm1iii,plotciii]

**iv) Expression of male tactic 1 reaches its maximum at intermediate levels of OSR (i.e., parabolic response curve).**

"Define response curve for male tactic 1 and male tactic 2:";

a=-0.25;"a = determines slope and orientation of curve";

b=-2;"f(–b) = y-max";

d=2;"d = 2 gives parabola";

e=1;"e = y-max";

m1iv=Clip[(a*(OSR+b)^d+e),{0,1}];"Clip function limits proportion of males expressing ART to 0–1";

m2iv=1-m1iv;"Proportion of males expressing tactic 1 + tactic 2 = 1";

"Female fitness costs associated with male interactions:";

c1=1;"c_1_ = female fitness cost associated with alternative male reproductive tactic 1";

c2=0.5;"c_2_ = female fitness cost associated with alternative male reproductive tactic 2";

"Male-female interaction rates:";

n=1;"Number of male-female interactions is constant = 1";

"Response curve for male tactic A (solid line):";

pm1iv=Plot[m1iv,{OSR,0,4},PlotStyle->{Thickness[0.02]}];

"Female cost curve when frequency of male-female interactions is constant = 1 (transparent surface):";

plotciv=Plot[(c1*m1iv+c2*m2iv)*n,{OSR,0,4},PlotStyle->{Thickness[0.012],Dotted}];

"Show response curves for both male reproductive tactics in one plot:";

Show[pm1iv,plotciv]

**Figure 2. Number of male interactions per female as a function of OSR and population density.**

"Clear all previous definitions:";

Clear["Global`*"]

"Define x- and y-axes for all Figure 2:";

xticks=Join[Table[{x,NumberForm[x,4],{0,0.02}},{x,0,4.5,1}],Table[{x,"",{0,0.01}},{x,0,4.5,0.5}]];

yticks=Join[Table[{y,NumberForm[y,4],{0,0.02}},{y,0,4.5,1}],Table[{y,"",{0,0.01}},{y,0,4.5,0.5}]];

"Define figure format for all Figure 2:";

SetOptions[Plot,BaseStyle->{FontFamily->"Helvetica",FontWeight->"Bold",FontSize->20},PlotRange->{{0,4},{0,4.45}},Axes->True,Frame->{True,True,False,False},FrameTicks->{xticks,yticks,None,None},FrameTicksStyle->Directive[18],FrameLabel->{"OSR","Number of male interactions

per female (n)"},ImagePadding->90,AspectRatio->0.9,ImageSize->400];

**i) The number of male interactions per female (*n*) varies with male density (ρm).**

"ρ = population density, total number of reproductively active individuals within encounter range of female during current time period; curves shown for three different population densities";

"Population density = 2 (solid line):";

ρ0=0;

n0=ρ0*(OSR/(OSR+1));

pi0=Plot[n0,{OSR,0,4},PlotStyle->{Thickness[0.03]}];

"Population density = 4 (dashed line):";

ρ2=2;

n2=ρ2*(OSR/(OSR+1));

pi2=Plot[n2,{OSR,0,4},PlotStyle->{Thickness[0.012],Dashed}];

"Population density = 6 (dotted line):";

ρ4=4;

n4=ρ4*(OSR/(OSR+1));

pi4=Plot[n4,{OSR,0,4},PlotStyle->{Thickness[0.012],Dotted}];

"Show curves for all three population densities in one plot:";

Show[pi0,pi2,pi4]

**ii) The number of male interactions per female (*n*) varies with operational sex ratio (OSR).**

"Curve is the same for all population densities";

nii=OSR;

pii=Plot[nii,{OSR,0,4},PlotStyle->{Thickness[0.02]}];

"Show curve in plot";

Show[pii]

**Figure 3. Variation in female fitness costs associated with OSR-dependent male ARTs. The solid surfaces represent female fitness costs when the number of male-female interactions is directly proportional to male density (ρ_m_ ) or the operational sex ratio (OSR). The transparent surfaces represent female fitness costs associated with male ARTs when male-female interaction rates are constant (n = 1).**

"Clear all previous definitions:";

Clear["Global`*"]

"Define global figure format for all Figure 2:";

SetOptions[Plot3D,BaseStyle->{FontFamily->"Helvetica",FontWeight->"Bold"},PlotRange->{{0,4.3},{0,4},{-1,4}},Axes->True,BoxStyle->Directive[Dashed],Ticks->{xticks,yticks,zticks},AxesLabel->{Framed["OSR",FrameStyle->None,FrameMargins->15],Framed["ρ",FrameStyle->None,FrameMargins->20],Framed["C_f_",FrameStyle->None,FrameMargins->20]},AxesEdge->{{-1,-1},{+1,-1},{-1,-1}},TicksStyle->Directive[FontSize->25,Thick],AxesStyle->FontSize->35,ImagePadding->88,AspectRatio->1,ImageSize->550];

"Define global x- and y-axes for all Figure 2:";

xticks=Join[Table[{x,NumberForm[x,4],{0,0.01}},{x,0,4,1}],Table[{x,"",{0,0.01}},{x,0,4,0.5}]];

yticks=Join[Table[{y,NumberForm[y,4],{0,0.002}},{y,0,4,1}],Table[{y,"",{0,0.01}},{y,0,4,0.5}]];zticks=Join[Table[{z,NumberForm[z,4],{0,0.002}},{z,-1,4,1}],Table[{z,"",{0,0.01}},{z,-1,4,0.5}]];

"Define global model parameters for all Figure 2:";

"Female fitness costs associated with male interactions:";

c1=1;"c_1_ = female fitness cost associated with alternative male reproductive tactic 1";

c2=0.5;"c_2_ = female fitness cost associated with alternative male reproductive tactic 2";

"Male-female interactions:";

n=1;"Number of male-female interactions is constant = 1";

ρm=ρ*(OSR/(OSR+1));"ρ_m_ = male density";

kρ=1;

bρ=0;

dρ=1;

nρ=kρ*ρm*dρ+bρ;"Number of male-female interactions is directly proportional to male density";

kOSR=1;

bOSR=0;

nOSR=kOSR*OSR+bOSR;"Number of male-female interactions is directly proportional to OSR";

**i) The expression of male tactic 1 does not vary with OSR (m_1_= m_2_= 0.5).**

"Proportion of males expressingt tactic 1 = proportion of males expressingt tactic 2 = 0.5";

m1i=0.5;

m2i=1-m1i;"Proportion of males expressing tactic 1 + tactic 2 = 1";

"Female cost curve when number of male-female interactions is constant = 1 (transparent surface):";

ploti=Plot3D[(c1*m1i+c2*m2i)*n,{OSR,0,4},{ρ,0,6},PlotStyle->None];

"Female cost curve when number of male-female interactions is directly proportional to male population density (solid surface in Ai):";

plotAi=Plot3D[(c1*m1i+c2*m2i)*nρ,{OSR,0,4},{ρ,0,6},PlotStyle->{Thickness[0.02]}];

"Female cost curve when number of male-female interactions is directly proportional to OSR (solid surface in Bi):";

plotBi=Plot3D[(c1*m1i+c2*m2i)*nOSR,{OSR,0,4},{ρ,0,6},PlotStyle->{Thickness[0.02]}];

Show[ploti,plotAi,ViewPoint->{1.527161876000964`,-2.8826863552358257`,0.8988303409578481`}]

Show[ploti,plotBi,ViewPoint->{1.527161876000964`,-2.8826863552358257`,0.8988303409578481`}]

**ii) Expression of male tactic 1 increases with OSR (i.e., positive linear response curve).**

"Define positive linear curve for male tactic 1, and inverse for male tactic 2:";

kii=0.25;"k = slope";

bii=0;"b = intercept";

m1ii=Clip[(kii*OSR+bii),{0,1}];"Clip function limits proportion of males expressing ART to 0–1";

m2ii=1-m1ii;"Proportion of males expressing tactic 1 + tactic 2 = 1";

"Female cost curve when number of male-female interactions is constant = 1 (transparent surface):";

plotii=Plot3D[(c1*m1ii+c2*m2ii)*n,{OSR,0,4},{ρ,0,6},PlotStyle->None];

"Female cost curve when number of male-female interactions is directly proportional to male population density (solid surface in Ai):";

plotAii=Plot3D[(c1*m1ii+c2*m2ii)*nρ,{OSR,0,4},{ρ,0,6},PlotStyle->{Thickness[0.02]}];

"Female cost curve when number of male-female interactions is directly proportional to OSR (solid surface in Bi):";

plotBii=Plot3D[(c1*m1ii+c2*m2ii)*nOSR,{OSR,0,4},{ρ,0,6},PlotStyle->{Thickness[0.02]}];

Show[plotii,plotAii,ViewPoint->{1.527161876000964`,-2.8826863552358257`,0.8988303409578481`}]

Show[plotii,plotBii,ViewPoint->{1.527161876000964`,-2.8826863552358257`,0.8988303409578481`}]

**iii) Expression of male tactic 1 decreases with OSR (i.e., negative linear response curve).**

"Define negative linear curve for male tactic 1, and inverse for male tactic 2:";

kiii=-0.25;"k = slope";

biii=1;"b = intercept";

m1iii=Clip[(kiii*OSR+biii),{0,1}];"Clip function limits proportion of males expressing ART to 0–1";

m2iii=1-m1iii;"Proportion of males expressing tactic 1 + tactic 2 = 1";

"Female costs when number of male-female interactions is constant = 1 (transparent surface):";

plotiii=Plot3D[(c1*m1iii+c2*m2iii)*n,{OSR,0,4},{ρ,0,6},PlotStyle->None];

"Female costs when number of male-female interactions is directly proportional to male population density (solid surface in Aii):";

plotAiii=Plot3D[(c1*m1iii+c2*m2iii)*nρ,{OSR,0,4},{ρ,0,6}];

"Female costs when number of male-female interactions is directly proportional to OSR (solid surface in Bii):";

plotBiii=Plot3D[(c1*m1iii+c2*m2iii)*nOSR,{OSR,0,4},{ρ,0,6},PlotStyle->{Thickness[0.02]}];

Show[plotiii,plotAiii,ViewPoint->{1.527161876000964`,-2.8826863552358257`,0.8988303409578481`}]

Show[plotiii,plotBiii,ViewPoint->{1.527161876000964`,-2.8826863552358257`,0.8988303409578481`}]

**iv) Expression of male tactic 1 reaches its maximum at intermediate levels of OSR (i.e., parabolic response curve).**

"Define parabolic curve for male tactic 1, and inverse for male tactic 2:";

a=-0.25;"a = determines slope and orientation of curve";

biv=-2;"f(–b) = y-max";

c=2;"c = 2 gives parabola";

d=1;"d = y-max";

m1iv=Clip[(a*(OSR+biv)^c+d),{0,1}];"Clip function limits proportion of males expressing ART to 0–1";

m2iv=1-m1iv;"Proportion of males expressing tactic 1 + tactic 2 = 1";

"Female costs when number of male-female interactions is constant = 1 (transparent surface):";

plotiv=Plot3D[(c1*m1iv+c2*m2iv)*n,{OSR,0,4},{ρ,0,6},PlotStyle->None];

"Female costs when number of male-female interactions is directly proportional to male population density (solid surface in Aiii):";

plotAiv=Plot3D[(c1*m1iv+c2*m2iv)*nρ,{OSR,0,4},{ρ,0,6}];

"Female costs when number of male-female interactions is directly proportional to OSR (solid surface in Biii):";

plotBiv=Plot3D[(c1*m1iv+c2*m2iv)*nOSR,{OSR,0,4},{ρ,0,6},PlotStyle->{Thickness[0.02]}];

Show[plotiv,plotAiv,ViewPoint->{1.527161876000964`,-2.8826863552358257`,0.8988303409578481`}]

Show[plotiv,plotBiv,ViewPoint->{1.527161876000964`,-2.8826863552358257`,0.8988303409578481`}]
